# Potential evolutionary impact of integrative and conjugative elements (ICEs) and genomic islands in the *Ralstonia solanacearum* species complex

**DOI:** 10.1038/s41598-020-69490-1

**Published:** 2020-07-27

**Authors:** Osiel Silva Gonçalves, Marisa Vieira de Queiroz, Mateus Ferreira Santana

**Affiliations:** Departamento de Microbiologia, Instituto de Bsiotecnologia Aplicada à Agropecuária (BIOAGRO), Universidade Federal de Viçosa, Viçosa, MG 36570-000 Brazil

**Keywords:** Genome evolution, Mobile elements

## Abstract

*Ralstonia solanacearum*, a soil-borne plant pathogen, encompasses a large number of strains known as *R. solanacearum* species complex (RSSC). Although it has been suggested that mobile genetic elements (MGEs) may play an important role in the RSSC genome, the evolutionary impact of these elements remains unknown. Here, we identified and analysed Integrative and Conjugative Elements (ICEs) and Genomic Islands (GIs) in the 121 genomes published for *Ralstonia* spp., including RSSC strains and three other non-plant pathogenic *Ralstonia* spp. Our results provided a dataset of 12 ICEs and 31 GIs distributed throughout *Ralstonia* spp. Four novel ICEs in RSSC were found. Some of these elements cover 5% of the host genome and carry accessory genes with a potential impact on the fitness and pathogenicity of RSSC. In addition, phylogenetic analysis revealed that these MGEs clustered to the same species, but there is evidence of strains from different countries that host the same element. Our results provide novel insight into the RSSC adaptation, opening new paths to a better understanding of how these elements affect this soil-borne plant pathogen.

## Introduction

The soil-borne bacterium *Ralstonia solanacearum* is one of the most devastating phytopathogens worldwide, responsible for bacterial wilt disease in more than 250 plant species^1,2^. Strains of *R. solanacearum* form a heterogeneous group of species that are divided into four phylotypes corresponding to their geographic origin. Recently, a taxonomic and nomenclatural update has proposed that the RSSC should encompass three distinct species: *R. pseudosolanacearum* (formerly phylotypes I and III), *R. solanacearum* (IIA and IIB), and *R. syzygii* (formerly phylotype IV and Blood Disease Bacterium)^3,4^.

*Ralstonia solanacearum* strains have a characteristic bipartite genome structure (chromosome and megaplasmid). In the genome of *R. pseudosolanacearum* GMI1000, a type strain, these replicons have a mosaic structure containing numerous mobile genetic elements (MGEs), signalling the potential for evolution through horizontal gene transfer (HGT)^5^. MGEs are efficient vectors for HGT that can contribute to bacterial genome evolution. In addition to MGEs encoding enzymes that mediate their inter or intracellular movement, these elements commonly encode accessory proteins that can confer important properties on the host cell^6^. Recently, as a result of the increase in bacterial genome sequences, it has been reporting the impact of MGEs in bacterial adaptation through the acquisition of novel genetic traits^7,8^. Furthermore, Genomic Islands (GIs) and Integrative and Conjugative Elements (ICEs) are recognized to contribute to bacterial genome adaptation, plasticity, and evolution^6^.

Genomic islands are genomic regions horizontally acquired, recognized by CG% content and codon usage bias that usually differ from the rest of the genome. They are often inserted near to tRNA genes and are flanked by 16–20-bp direct repeats^9^. GIs harbour components of MGEs that may have been implicated in mobilizing DNA^10^. Furthermore, these regions often carry genes conferring a selective advantage to the host bacterium, which, depending on their gene functions, may be referred to as pathogenicity, symbiosis, metabolic, fitness, or resistance islands^9,11^. ICEs are chromosomal, self-transmissible mobile elements that can also be passively propagated during chromosomal replication, cell division, or horizontal transfer via conjugation^12^. This genetic element has a modular structure such as a recombination module, a conjugation module, and a regulation module, which together control and ensure the excision and transfer of the ICEs. The acquisition of ICEs and GIs may be attributed to a great source of genetic variation and ecological adaptation to certain plant pathogens^13,14^. However, how these elements impact the fitness and pathogenicity of RSSC strains remains unclear. To close this knowledge gap, we conducted genome mining and comparative genomic analysis based on the repertoire of ICEs and GIs in the 121 *Ralstonia* spp. genomes.

## Results

### The majority of ICEs and GIs are preferentially associated with *R. pseudosolanacearum* strains

**Figure 1:**
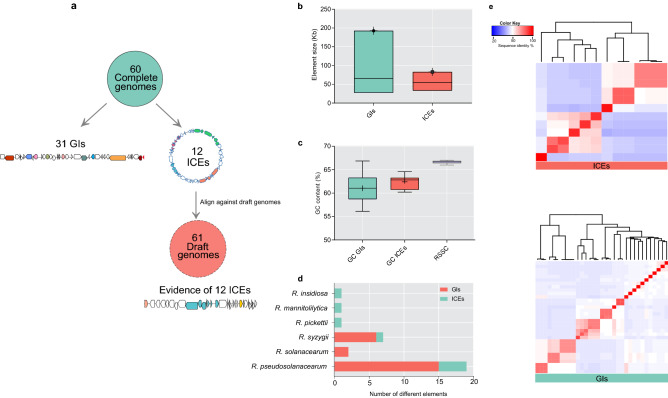
Comparison between GIs (green) and ICEs (orange). (**A**) Schematic representation of the identified elements in *Ralstonia* spp. genomes. (**B**) Bar chart representing the size distribution of the elements. Average sizes: GIs (65.5 kb), ICEs (54.4 kb). (**C**) Bar chart showing the distribution of the GC% content between GIs, ICEs, and *Ralstonia* spp. (**D**) Distribution of GIs and ICEs in the genomes of *Ralstonia* spp. (**E**) Heatmap visualizing the pairwise identity matrix of ICEs and GIs among the RSSC and *Ralstonia* spp. genomes. The colours represent, as indicated in the legend, the average similarity values for the sequences.

To evaluate the evolutionary impact of ICEs and GIs on *Ralstonia* spp., we first characterized the composition and distribution of these two elements. As shown in Fig. 1a, 12 ICEs were mapped in the complete genomes, and evidence of 12 ICEs was found in the draft genomes. 31 putative GIs were found distributed throughout the 60 genome sequences (Fig. 1a). Due to the mosaic nature and extreme variety of GIs, we decided not to perform a local alignment against draft genomes. The size of GIs ranged from 27 to 192 kb, and the ICEs ranged from 41 to 83 kb. The average size of GIs was slightly larger than that of ICEs (65 kb vs. 54 kb) (Fig. 1b). Both ICEs and GIs were found to have GC content lower than their host genomes (66% CG content), varying from 56 to 65 and 60 to 65, respectively (Fig. 1c). None of the ICEs or GIs were identified in the megaplasmid sequences according to our criteria. Also, no GIs were found in these three non-plant pathogenic strains.

**Figure 2:**
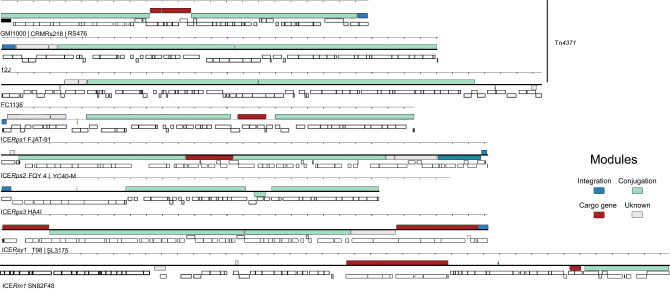
Mauve alignment of ICEs from *Ralstonia* spp. genomes. Coloured blocks represent co-linear blocks, and the histogram inside each box shows the average level of conservation in that region. Completely white areas were not aligned and contained sequence elements specific to a particular ICE.

Overall, GIs and ICEs were distributed throughout the complex, being preferentially associated with the *R. pseudosolanacearum* strains (Fig. 1d). These elements together constitute 4–5% of its chromosome (data not shown). A point of note, multiple GIs were found for *R. pseudosolanacearum*. The strains GMI1000, CRMRs218, and RS476 host GIRps01 and GIRps02 in their chromosome insert in the tRNA^Ala^ gene in the same position (Supplementary Table S1). Also, the Tn*4371* ICE family observed in *R. pseudosolanacearum* GMI1000^5^ was found in the chromosome of CRMRs218 and RS476, sharing 100% nucleotide identity. We also mapped Tn*4371* in the genomes of *R. pickettii* and *R. insidiosa*^15^. In addition, we identified ICE*Rm1*, a novel ICE with 83 kb long, in the chromosome of *R. mannitolilytica* SN82F48 (Fig. 2, Supplementary Table S2).

Notable variable genes encoded by the ICEs revealed a clear difference between RSSC strains and non-plant pathogenic *Ralstonia* spp. Most RSSC ICEs coding for a repertoire of genes with potential impact on RSSC fitness and pathogenicity, e.g. hydrolase proteins, stress response, and aromatic compound metabolism. In contrast, ICEs found in non-plant pathogenic *Ralstonia* spp., mostly emerging opportunistic pathogens, code for antibiotic resistance genes (Beta-lactam antibiotics), and fatty acid and sugar metabolism (Supplementary Table S2).

**Figure 3:**
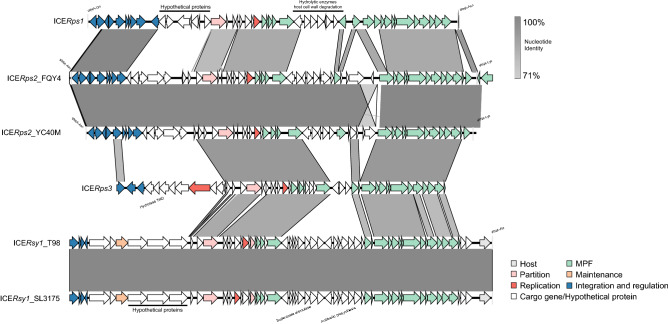
Schematic representation of several novel ICE structures identified in the RSSC genomes. Genes are represented by arrows with different colours according to their functions. Modules are color-coded, and their functional designations are labelled in the figure. Abbreviations for MPF, Mating Pair Formation.

Altogether, 23 different GIs and 6 different ICEs were found in our dataset (Fig. 1e, Supplementary Table S1, and Supplementary Table S2). These elements share a low degree of nucleotide sequences with other species, indicating an exclusive association with *Ralstonia* spp. strains. Different from ICEs, the GIs lack the key functions of mobilisation, and their regulation. Another important observation from our analysis is that 63% of ICEs use tRNA as an insertion site. Details of the elements are listed in Supplementary Table S1 and Supplementary Table S2. ICEs show collinear syntenic blocks, representing the core modular structure, which displayed a high degree of similarity between these ICEs (Fig. 2). Modular genes encode for mobilization proteins (e.g. VirD4), mating pair formation (MPF) (e.g. TrbB, TrbC, TrbD, TrbE, TrbF, TrbG, TrbI), secretion system proteins (e.g. VirD2, ATPases), replication proteins (e.g. RepA), maintenance protein (e.g. TA system), integrase/excisionase, and others (e.g. phage-related proteins) (Figs. 2 and 3).

### Identification of the four novel ICEs in RSSC

Out of 12 ICEs mapped, our analysis revealed four novels ICEs (ICE*Rps1*, ICE*Rps2*, ICE*Rps3*, ICE*Rsy1*) identified in six RSSC strains (Fig. 3). These four ICEs are only related to *Ralstonia* spp. and share a low degree of nucleotide identity (< 50%) with other ICEs. *R. pseudosolanacearum* has three ICEs integrated into the chromosomes of the three strains. ICE*Rps1* is a 51 kb long ICE found integrated into the tRNA^Lys^ gene in the strain FJAT-91. The most notable feature carried by ICE*Rps1*, encodes hydrolase proteins. ICE*Rps2* is a 60 kb long ICE found next to the tRNA^Lys^ gene in the chromosome of two *R. pseudosolanacearum* strains FQY_4 and YC40-M. Accessory genes carry by ICE*Rps2* have very diverse functions, including stress response, membrane transport, sugar metabolism, and aromatic compound metabolism. ICE*Rps3* is a 46 kb long ICE identified in the chromosome of *R. pseudosolanacearum* HA4I. ICE*Rps3* is integrated in the tRNA adenosine and carries accessory genes encode for signal transduction proteins, hydrolytic enzymes, and proteins related to transferase activity substrates (Fig. 3).

ICE*Rsy1* is a 60 kb long ICE found in the chromosome of two *R. syzygii* strains T98 and SL3175. This ICE is integrated into the *guaA* gene in the same position for both strains. ICE*Rsy1* encodes proteins involved in stress response, particularly oxidative stress, which may be involved in the detoxification of the active oxygen species produced by infected plants, but also proteins related to aromatic compound metabolism, and storage of nutritious substrates (Fig. 3).

All ICEs contain conserved features of conjugative elements, including open reading frames (ORFs) encoding putative relaxase belonging to the MOB_P_/MOB_H_ family, type-IV coupling proteins (T4CP), and the type-IV secretion system (T4SS) (Supplementary Table S3 and Supplementary Table S4). Details of the conserved features and accessory genes found in the ICEs are listed in Supplementary Table S2.

### Functional repertoires of genes found in ICEs and GIs

**Figure 4:**
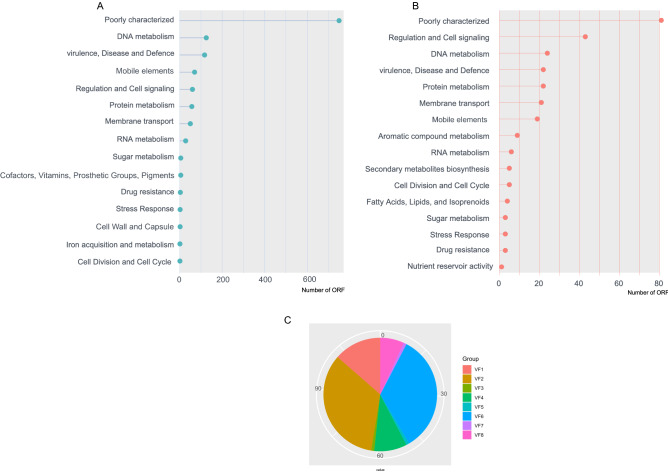
Functional comparisons of the ORFs carried by GIs (green) and ICEs (orange) from *Ralstonia* spp. genomes. (**A**) Representation of functional categories in GIs to the host chromosome. (**B**) Representation of functional categories in ICEs to the host chromosome. (**C**) Distribution of eight classes of virulence factors in GIs. VF1 (Adhesion/Surface proteins), VF2 (Hydrolytic enzymes/Host cell wall degradation), VF3 (Plant hormones and signalling molecules), VF4 (Potential Type III secretion-dependent effectors), VF5 (Resistance to oxidative stress), VF6 (Secretion system), VF7 (Toxins) and VF8 (Type III secretion system and secreted effectors).

The presence of ICEs and GIs in the bacterial host can confer selective advantages because these genomic regions often encode additional functions beyond what is essential for bacterial growth^16,17^. We then investigated whether fitness and pathogenicity genes can be linked to these two elements. We searched for the repertoires of genes in our dataset. The most prevalent ORFs correspond to hypothetical proteins, which are commonly found in GIs and ICEs^18,19^. In total, we located 1337 putative ORFs in GIs and 274 ORFs in ICEs. Most GIs and ICEs encode proteins characteristic of other mobile elements, including prophages, transposable elements, and plasmids-related proteins. Other major cluster was based on protein metabolism, which was assigned to several set subgroups, including transferase activity, transaminase activity, catalytic activity, and ATP binding. Also, we found in the GIs genes coding for proteins involved in cofactors, vitamins, prosthetic groups, and pigments. ORFs involved in iron acquisition and metabolism, sulphur metabolism, cell wall and capsule, cell division and cell cycle, and secondary metabolism were found (Fig. 4a, b).

We observed that virulence, disease and defence group was systematically more frequent in GIs and to a lesser extent ICEs. In subsequent analysis, we grouped the GIs virulence ORFs into eight classes (VF1 to VF8) corresponding, respectively, to Adhesion/Surface proteins, Hydrolytic enzymes/Host cell wall degradation, Plant hormones and signalling molecules, Potential Type III secretion-dependent effectors, Resistance to oxidative stress, Secretion system, Toxins, and Type III secretion system (T3SS)/secreted effectors, according to Salanoubat et al.^5^. In Fig. 4c, we demonstrate the distribution of each virulence class. The VF2 (Hydrolytic enzymes/Host cell wall degradation) and VF6 (Secretion system) were found to be the most prevalent classes throughout the GIs, 34% (n = 40) and 34% (n = 40), respectively. Another class that we highlight is VF8 (Type III secretion system (T3SS)/secreted effectors) found in 6% (n = 8) of GIs. This class, essential for pathogenicity, includes genes coding for Type III effector proteins for Ript, Skwp 4, PopP2, and PopP1. The most frequent virulence factor in ICEs corresponds to the Hydrolytic enzymes/Host cell wall degradation class (22/31).

### Evolutionary relationships between GIs and ICEs throughout the RSSC

**Figure 5:**
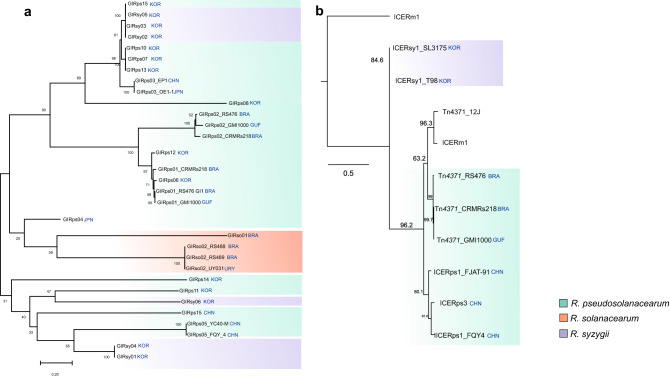
Maximum likelihood phylogeny analysis based on conserved markers to infer relationships between ICEs and GIs. (**A**) Phylogenetic analysis of Integrases/recombinase (*int*/XER-Ser-Tyr) of GIs showing clusters according to species. (**B**) Phylogenetic analysis of the conjugative gene (*tbrB*) of ICEs showing clusters according to species of the *Ralstonia* spp. strains. The tree was generated with MEGA-X software using the Maximum Likelihood and the algorithm of Jukes and Cantor with 1000 bootstrap re-samplings. Bootstrap values (≥ 50%) are shown beside each node. Codes in blue represent the names of countries according to The International Organization for Standardization (ISO) 3166: BRA, Brazil; CHN, China; GUF, French Guyana; JPN, Japan; KOR, Korea; PER, Peru; URY, Uruguay.

Reconstruction of RSSC GIs and ICEs phylogeny was based on the nucleotide sequences of *int* and *trbB*, respectively. The phylogeny shows several clusters containing highly related GIs (Fig. 5a) and ICEs (Fig. 5b), which were identified to belong to the same species. However, we noticed many noteworthy aspects. We found that *R. pseudosolanacearum* strains from Brazil (RS 476 and CMRs218) and French Guyana (GMI1000) share the same GIs and ICEs sequences. The sequences of these GIs (GIRps01 and GIRps02) and ICEs (*Tn4371*) shared by these three strains have at least 96% and 99% identity with 99% coverage, respectively (data not shown). The same pattern was observed in the GIRps03 hosted by the strains EP1 (China) and OE1 (Japan). GIRso02 sequences shared by *R. solanacearum* strains from Brazil (RS 488 and RS 489) and Uruguay (UY031) (Fig. 5a), with 100% sequence identity with 99% coverage. ICE-associated *trbB* forms a separate clade within the *R. pseudosolanacearum* strains, with distinct divergent clades related to the geographic distribution (American strains and Asian strains) (Fig. 5b). In summary, these results demonstrate a phylogenetic distribution of GI-encoded integrases and ICE-encoded trbB with representatives from most RSSC strains.

## Discussion

*Ralstonia solanacearum* is a useful model for exploring the evolution patterns in closely connected strains due to the formation of emerging ecotypes (highly host-adapted strains) adapting to local environmental conditions^20,21^. Herein, we provide genomic insights into this diversified adaptation based on the repertoire of ICEs and GIs in a large set of strains within the RSSC. We conducted genome mining and comparative genomic analysis of 121 sequenced *Ralstonia* spp. Our analysis mapped a total of 43 integrative mobile regions. The number of GI elements can be even higher since 61 genomes of *Ralstonia* spp. are in draft assemblies (split into multiple contigs), and it has become a daunting task to identify^22^, due to the extreme variety of gene content carrying by GIs^23^, and the natural competence of *R. solanacearum* for DNA transformation, which confers its genomic plasticity^24^. In addition, ICEs and GIs are often known to be absent in several closely related genomes^25^. We compared the composition and distribution of the ICEs and GIs and evaluated their evolutionary impact. These elements are characterized by their large size (> 10 kb) and a different GC content compared with the rest of the genome^23,25^. We found that ICEs and GIs constitute a large fraction of the *Ralstonia* genomes, varying in size and GC content. Especially in the case of the ICEs, GC content tends to become more similar to the compositional signature of their host, suggesting that ICEs are undergoing a domestication process^26,27^.

None of the ICEs or GIs were found in the megaplasmid sequences according to our criteria. This may be explained by the number of tRNA genes found on the chromosome and megaplasmid, as the most predominant ICEs and GIs were preferentially associated with tRNA and tmRNA genes. We found an average of 55 tRNA in the chromosome and three tRNA genes in the megaplasmid (data not shown). Therefore, ICEs and GIs associated with tRNA and tmRNA appear to be good indicators for site-specific integration and HGT in RSSC^28^. Most identified ICEs and GIs were present in the *R. pseudosolanacearum*. This observation could be because that *R. pseudosolanacearum* covers a significant number of genomes analysed in this study. We also revealed that GIs from this species occupied a large fraction of its chromosomes. *R. pseudosolanacearum* and *R. solanacearum* are recognized to have been arranged in clonal complexes worldwide, which may also contribute to this preference^29^. Consequently, Safni et al.^4^ suggest further work to better understand and propose the designation of subspecies for one or both species, especially for *R. pseudosolanacearum*, where a clear geographical division exists. Here, we have demonstrated that the identified elements cluster to the same species, but there is evidence of strains from different countries in America (Brazil, French Guyana, and Uruguay) host the same element. This evidence is supported by Wicker et al.^30^, which have reported that formerly phylotype II, from America, presented evidence of gene flow by analysing recombination patterns. Furthermore, our results may contribute to the hypothesis that a common ancestor of all phylotype II strains probably emerged from Brazil^31^.

Besides the ICEs already reported, e.g. Tn*4371* ICE family in the *R. pseudosolanacearum* strain GMI1000^5^, *R. pickettii*, and *R. insidiosa*^15^, we identified four novel ICEs in the RSSC genomes. Despite the mosaic structure shown by these novel ICEs, we have demonstrated a collinearity identity between them. In general, this identity represents the core modular structure-related functions, including conjugation, recombination, and regulation^12,18^. ICEs and GIs were analysed, and further searches were carried out for cargo genes. We found genes of putative function related to bacterial fitness. Among these, we highlight the cluster of genes coding for protein metabolism, cofactors, vitamins and pigments, genes involved in iron acquisition, and sulphur metabolism. These subsets of functional clusters may help the RSSC to thrive in the plant environment, contributing to its diversified adaptation^32^.

Several putative genes coding for virulence factors were found. Next, we represented in eight classes corresponding to adhesion/surface proteins, hydrolytic enzymes/host cell wall degradation, plant hormones and signalling molecules, potential type III secretion-dependent effectors, resistance to oxidative stress, secretion system, toxins, T3SS and secreted effectors. These eight classes were represented in most GIs identified, which shows their widespread dissemination throughout these genomic elements. We also found that 8% of GIs encode T3SS effectors, which are recognized as essential for RSSC pathogenicity^20^. T3SS is responsible for the synthesis and also the injection of a variety of effector proteins into the plant cells during the infection stage, which can cause disease in a susceptible host or a hypersensitive response in the resistant hosts^33,34^.

*Ralstonia solanacearum* pathogenicity is also determined by several other virulence factors, such as plant cell wall-degrading enzymes. These genes are secreted across the outer membrane by the type II secretion system, recognized as essential for the colonization phase^35^. Here, we identified a collection of GIs and ICEs carrying important hydrolytic enzymes that are secreted by this system. During the infection phase, *R. solanacearum* faces a variety of reactive oxygen species (ROS) made by plants, and the expression of genes that confer resistance to oxidative stress allows the bacteria to tolerate this oxidative environment. Not surprisingly, mutants lacking genes that confer resistance to oxidative stress, e.g. peroxidase and regulator of oxidative stress genes, significantly reduce virulence^36^. Also, evidence of positive regulation of phytohormone production, e.g. ethylene and auxin, during interaction with plants might contribute to the virulence of *R. solanacearum*, but its role is still poorly understood^34^. It should be noted that our results show a great variety of *R. solanacearum* virulence factors such as gene cargo in ICEs and GIs, which might provide a selective phenotype for this important pathogen. In summary, these results demonstrate a series of putative ORFs that may be involved in the RSSC’s diversified adaptation to environmental conditions and genes for their pathogenicity.

## Methods

### Data collection

106 RSSC and 15 *Ralstonia* spp. genome sequences were retrieved from the National Center for Biotechnology Information (NCBI, https://www.ncbi.nlm.nih.gov/genome) (Supplementary Table S5), in August 2019. We used the classification of replicons as chromosomes and megaplasmid as provided in the GenBank files to perform our analyses.

### Detection and delimitation of ICEs

We used three strategies to identify ICEs. First, standard BLASTn^37^ searches against the ICEberg database version 1.0 (https://db-mml.sjtu.edu.cn/ICEberg/)^38^, only matches with the E-value ≤ 10^–5^ and sequence coverage ≥ 50% were retained. Second, we found novel ICEs searching for conserved conjugation and DNA processing genes in annotated genomes deposited in the NCBI database. Relaxase MOB family assignments were made using the MOBscan server (https://castillo.dicom.unican.es/mobscan/) (Supplementary Table S3)^39^. The genomes were inspected for MGE-encoding relaxases, T4CP, and the T4SS gene cluster using the oriTfinder tool (https://bioinfo-mml.sjtu.edu.cn/oriTfinder) (Supplementary Table S4)^40^. An element was considered as conjugative when it contained the following components of the conjugative system: relaxase, T4CP, and the presence of T4SS gene cluster^41,42^. Finally, we built a local database with 166 conserved proteins from previously identified ICEs for *Ralstonia* spp. (Supplementary Table S6). Next, we performed BLASTp alignment^43^ against complete and draft genomes of *Ralstonia* spp. to detect conserved features indicative of conjugative elements. Hits were regarded as significant when their e-value was 10^−5^ and their alignment covered at least 30% of the protein profile.

We analysed core genes flanking these loci to provide the upper bounds for the limits of the ICEs. Similar to Cury et al.^18^, we overlaid the presence of genes of the conjugation system, and GC content to delimit the ICEs. Predicted ICEs were inspected for DRs that define the boundaries of the element. The complete nucleotide sequences of the ICEs, in GenBank format of corresponding records, were imported into the GENEIOUS 11.1.5 program (Biomatters Ltd) to help delimit genomic regions flanking the elements. We used the annotations of the GenBank files for our predicted ICEs and categorized the CDS in clusters of orthologous groups (COGs) with COG v1.0 against NCBI preformatted CDD (conserved domains database) with an E-value cutoff of 10^−5^. ICEs were named in accordance with the nomenclature system proposed by Burrus et al.^44^.

### Detection of GIs

GIs were predicted by the interface Island Viewer 4^45^ with default parameters using the following GI prediction methods: IslandPick, IslandPath-DIMOB, SIGI-HMM, and Islander. For accurate analysis of GIs, only elements detected by the three methods (IslandPick, IslandPath-DIMOB, SIGI-HMM) were considered. These three methods use a comparative genomic prediction method to develop stringent data sets of GIs and non-GIs^25^, abnormal sequence composition and presence of genes functionally related to mobile elements^46^, and measurements of dinucleotide bias, respectively^47^. The GIs were then manually filtered to avoid false-positive and negative data sets according to the following criteria: (1) presence of mobility genes (e.g., integrases, recombinases, and transposases); (2) proximal structural RNA (tRNA and tmRNA); (3) atypical GC% content; and (4) presence of factors for virulence, metabolism, antibiotic resistance, or symbiosis. Predicted GIs were extracted and systematically annotated using the RAST server (https://rast.nmpdr.org/)^48^. Direct repeats and insertion sequences were manually identified in annotated sequences using GENEIOUS. GIs were inspected for Relaxase MOB family using the MOBscan server (https://castillo.dicom.unican.es/mobscan/)^39^, for T4CP, and the T4SS gene cluster using the oriTfinder tool (https://bioinfo-mml.sjtu.edu.cn/oriTfinder)^40^. Next, different genomic sequence signatures (e.g., G + C content, GC-skew, codon usage) were also measured using GENEIOUS plugins. CDS were categorized into COGs with COG v1.0 against NCBI preformatted CDD with an E-value cutoff of 10^−5^. GIs were named as follows: GI + three-letter acronyms of the species + sequential number.

### Virulence-associated genes in ICEs and GIs

We performed a BLASTp (using parameters e-value ≤ 10^–5^ and amino acid identity > 30%) in the following database to analyse GIs and ICEs carrying virulence cargo genes: Virulence Factors Database (VFDB, https://www.mgc.ac.cn/VFs/)^49^; Pathogen–Host Interactions database (PHI-base, https://www.phi-base.org)^50^; Ralsto T3E database (https://www.ralsto-T3E.org)^51^; The Pathogenicity Island Database (PAIDB, https://www.paidb.re.kr^52^; Type III Secretion System Database (T3SEdb, https://www.effectors.bic.nus.edu.sg/T3SEdb)^53^.

### Element Comparisons

Integrases/recombinase (*int*/XER-Ser-Tyr) and conjugative gene (*tbrB*) were used as markers to infer phylogenetic relationships between GIs and ICEs, respectively. Nucleotide sequences were aligned using ClustalW^54^. After the assessment of the best nucleotide substitution model using jModeltest version 2.1.10^55^, a Maximum Likelihood phylogenetic tree was constructed in MEGA X (1000 bootstrap replicates)^56^ using Jukes and Cantor as nucleotide substitution model.

For colinear-gene analysis, the predicted ICEs were analysed for gene content and extracted from the genome to construct multiple sequence alignments using the Mauve software^57^. ICEs comparison was generated with Easyfig^58^.

## Supplementary information

Supplementary file1 (PDF 385 kb)

## Data Availability

All data generated or analysed during this study are included in this published article (and its supplementary information files).
