# Supplementary material for: Potential evolutionary impact of integrative and conjugative elements (ICEs) and genomic islands in the Ralstonia solanacearum species complex
Source: Sci Rep. 2020 Jul 27;10:12498. doi: 10.1038/s41598-020-69490-1 (PMC7385641; doi:10.1038/s41598-020-69490-1)
Supplement: Supplementary file 1 — Supplementary file1 (PDF 385 kb) [file 41598_2020_69490_MOESM1_ESM.pdf]

Supplementary Information

Potential evolutionary impact of Integrative and Conjugative Elements (ICEs) and Genomic Islands in the Ralstonia solanacearum species complex

Osiel Silva Gonçalves, Marisa Vieira de Queiroz, and Mateus Ferreira Santana

Departamento de Microbiologia, Instituto de Biotecnologia Aplicada à Agropecuária (BIOAGRO), Universidade Federal de Viçosa, Viçosa, MG, 36570-000, Brazil.

Supplementary Tables and Figures

Supplementary Table S1. Characteristics and coordinates of Genomic Islands identified in the *Ralstonia* spp. genomes.

Supplementary Table S2. Characteristics and coordinates of Integrative and conjugative element identified (ICEs) in the *Ralstonia* spp. genomes

Supplementary Table S3. Relaxase MOB families identified in the genome *Ralstonia* spp.

Supplementary Table S4. *Ralstonia* spp. genomes coding T4CP and T4SS.

Supplementary Table S5. *Ralstonia* spp. genomes used in this work.

Supplementary Table S6. Conserved proteins used to identify Integrative and Conjugative Elements.

Table S1. Characteristics and coordinates of Genomic Islands identified in the chromosome of *Ralstonia* spp.

Genomic island	Host	Length	coordinates	GC% content	Number of predicted ORFs	Insertion site	GenBank accession number
R. pseudosolanacearum							
GIRps01	GMI1000	94.3 Kb	3417490-3511839	63.3	96	tRNA-Ala	AL646052.1
GIRps02		80.7 Kb	872116-943347	64.2	79	tmRNA	
GIRps01	RS476	94,3 Kb	3417490-3511839	63.3	96	tRNA-Ala	CP021762.1
GIRps02		71.2 Kb	872131-943355	64.2	79	tmRNA	
GIRps01	CRMRs218	94,3 Kb	3417490-3511839	63.3	96	tRNA-Ala	CP021764.1
GIRps02		80.7 Kb	872131-943355	64.2	79	tmRNA	
GIRps03	EP1	60.2 Kb	1234176-1294537	56.1	61	tRNA-Ser	CP015115.1
GIRps03	OE1-1	35.3 Kb	3214582-3249933	58.4	35	tRNA-Thr	CP009764.1
GIRps04		45.3 Kb	2553544-2598873	58.4	37	tRNA-Ser	
GIRps05	FQY_4	48.5 Kb	3055877-3104433	59.1	48	tRNA-Thr	CP004012.1
GIRps05	YC40-M	38.9 Kb	1726826-1765845	64.7	33	tRNA-Arg	CP015850.1
GIRps06	T60	68.9 Kb	2888558-2957522	61.8	64	tRNA-Ser	CP022768.1
GIRps07	T42	57.6 Kb	1190504-1248147	57.6	56	tRNA-Ser	CP022772.1
GIRps08	SL3822	71.1 Kb	1205679-1276801	59.7	56	tRNA-Ser	CP022780.1
GIRps09	SL3755	192.5 Kb	2726603-2919152	62.4	161	tmRNA	CP022782.1
GIRps10	SL3730	85.4 Kb	1190367-1275794	61.6	81	tRNA-Ser	CP022784.1
GIRps11	SL3103	56.2 Kb	205572-261836	62.6	57	tRNA-Ala	CP022790.1
GIRps12		78.6 Kb	2534222-2612828	62.9	75	tmRNA	
GIRps13	SL2729	94.2 Kb	1190420-1284664	62	83	tRNA-Ser	CP022792.1
GIRps14		48.5 Kb	597355-645927	59.1	48	tRNA-Thr	
GIRps15	KACC10722	68.6 Kb	1100412-1169028	58.7	64	tRNA-Ser	CP014702.1
R. solanacearum							
GIRso01	IBSBF1503	27.5 Kb	2617958-2645495	56.8	24	tRNA-Met	CP012943.1
GIRso02	RS488	57.4 Kb	1201498-1258905	56.8	58	tRNA-Ser	CP021652.1
GIRso02	UY031	57.4 Kb	1201506-1258913	56.8	58	tRNA-Ser	CP012687.1
GIRso02	RS489	56.0 Kb	1202826-1258883	56.8	56	tRNA-Ser	CP021766.1
R. syzygii							
GIRsy01	T82	43.2 Kb	2363558-2406806	60.2	39	tRNA-Lys	CP022763.1
GIRsy02	T95	68.6 Kb	1107972-1176588	58.7	64	tRNA-Ser	CP022761.1
GIRsy03	T51	68.6 Kb	1053922-1122546	58.7	64	tRNA-Ser	CP022770.1
GIRsy04	SL2312	41.1 Kb	2363523-2404879	60	36	tRNA-Lys	CP022796.1
GIRsy05	SL2064	71.0 Kb	1107930-1179022	59.0	62	tRNA-Ser	CP022798.1
GIRsy06	SL3175	46.6 Kb	2672173-2718819	60.6	48	pdhA	CP022788.1

Table S2. Characteristics and coordinates of Integrative and conjugative element ICEs) identified in the chromosome of *Ralstonia* spp.

Name	Host	Length	%GC	Coordinates	Integrase	Site integration	Relaxase	Cargo genes encode for	GenBank accession number	Reference
R. pseudosolanacearum										
Tn*4371*	GMI1000	45Kb	63.2	2780151-2825764	Ser	*purM*	MOB$_P$	Hydrolase proteins, coenzyme related to transport and metabolism	AL646052.1	Boucher et al., 1985
Tn*4371*	RS476	45Kb	63.2	2780170-2825773	Ser	*purM*			CP021762.1	In this study
Tn*4371*	CRMRs218	45Kb	63.2	2780419-2826032	Ser	*purM*			CP021764.1	In this study
ICE*Rps1*	FJAT-91	51.2 Kb	60.9	1283877-1335170	Tyr	tRNA^{Lys}	MOB$_P$	Hydrolase proteins	CP016612.1	In this study
ICE*Rps2*	FQY_4	60.3 Kb	63	1113417-1173796	Tyr	tRNA^{Lys}	MOB$_P$	Stress response, membrane transport, sugar metabolism and aromatic compound metabolism	CP004012.1	In this study
ICE*Rps2*	YC40-M	55 Kb	62.3	2532466-2588165	Tyr				CP015850.1	In this study
ICE*Rps3*	HA4I	46.8 Kb	62.2	1709954-1756828	Tyr	tRNA adenosine	MOB$_P$	Signal transduction protein, hydrolytic enzyme and protein related to transferase activity	CP022481.1	In this study
R. syzygii										
ICE*Rsy1*	T98	60.2 Kb	60.2	1925451-1985747	-	*guaA*	MOB$_P$	Hydrolase activity, protein related to stress response, aromatic compound metabolism, and storage protein of nutritious substrates	CP022759.1	In this study
ICE*Rsy1*	SL3175	60.2 Kb	60.2	1925467-1985763	-	*guaA*			CP022788.1	In this study
R. pickettii										
Tn*4371*	12J	54.2 Kb	64.6	2715217-2769400	Tyr	*panC*	MOB$_P$	Beta lactamase resistance, fatty acid metabolism and sugar metabolism	CP001068.1	Ryan et al., 2009
R. mannitolilytica										
ICE*Rm1*	SN82F48	83 Kb	63.5	1635815-1718887	Phage	tRNA^{Gly}	MOB$_H$	Chromate resistance, secondary metabolites biosynthesis, stress metabolism and sugar metabolism	CP010799.2	In this study
R. insidiosa										
Tn*4371*	FC1138	67 Kb	65.4	1314259-1381324	Phage	tRNA^{Pro}	MOB$_P$	Beta lactamase resistance, hydrolase activity and secretion system	CP012605.1	Ryan et al., 2009

Table S3. Relaxase MOB families identified in the genome *Ralstonia* spp.

Strain	Protein code	Relaxase	ICE
Po82	lcl|CP002819.1_prot_AEG70128.1_2824	MOBV	No
UY031	lcl|CP012687.1_prot_ALF88732.1_2331	MOBP	No
UW163	lcl|CP012939.1_prot_AMP68267.1_321	MOBV	No
IBSBF1503	lcl|CP012943.1_prot_AMP75679.1_2339	MOBV	No
RS488	lcl|CP021652.1_prot_ATI28168.1_2349	MOBP	No
RS489	lcl|CP021766.1_prot_ATJ86920.1_2334	MOBP	No
CFBP2957	-	-	No
K60	-	-	No
UW551	OYQ05424.1	MOBP	No
GMI1000	lcl|AL646052.1_prot_CAD16308.1_2599	MOBP	Yes
OE1	-	-	No
FJAT-1458	-	-	No
EP1	lcl|CP015115.1_prot_API74101.1_1127	MOBP	No
FJAT91	lcl|CP016612.1_prot_ARS55733.1_1226	MOBP	Yes
CQPS	lcl|CP016914.1_prot_ASL74286.1_2391	MOBP	No
RSCM	lcl|CP025985.1_prot_AUS41341.1_648	MOBF	No
	lcl|CP025985.1_prot_AUS43460.1_3087	MOBF	No
FQY	lcl|CP004012.1_prot_AGH83629.1_1108	MOBP	Yes
SEPPX05	cl|CP021448.1_prot_ARU20787.1_341	MOBF	No
	lcl|CP021449.1_prot_ARU24616.1_4170	MOBP	No
SL3103	-	-	No
SL2330	lcl|CP022794.1_prot_AXW70161.1_552	MOBF	No
T117	-	-	No
T78	cl|CP022765.1_prot_AXV94622.1_571	MOBQ	No
	lcl|CP022767.1_prot_AXV99181.1_3664	MOBP	No
T25	lcl|CP023014.1_prot_AXW14050.1_532	MOBF	No
SL3755	lcl|CP022782.1_prot_AXW37295.1_538	MOBF	No
SL3730	lcl|CP022784.1_prot_AXW44073.1_2785	MOBP	No
SL3130	lcl|CP022784.1_prot_AXW44073.1_2785	MOBP	No
SL2729	lcl|CP022792.1_prot_AXW67413.1_2784	MOBP	No
T110	lcl|CP023012.1_prot_AXV72975.1_534	MOBF	No
HA4-1	lcl|NZ_CP022481.1_prot_WP_111374838.1_659	MOBP	NO
	lcl|NZ_CP022481.1_prot_WP_111374384.1_1532	MOBP	Yes
	lcl|NZ_CP022483.1_prot_WP_164775032.1_5160	MOBP	Plasmid
T60	-	-	No
T42	lcl|CP022772.1_prot_AXW11513.1_2785	MOBP	No
SL3882	-	-	No
SL3822	-	-	No
SL3300	-	-	No
KACC10709	-	-	No
KACC10722	-	-	No
CMR15	lcl|FP885893.1_prot_CBJ36129.1_5122	MOBF	Plasmid
Rs-10-244	-	-	No
Rs-09-161	lcl|NZ_CM002757.1_prot_WP_161780514.1_2226	MOBF	No
	lcl|NZ_JHBO01000207.1_prot_WP_082240131.1_5008	MOBQ	No
YC40M	lcl|CP015850.1_prot_ANH33580.1_2387	MOBP	Yes
RS476	lcl|CP021762.1_prot_AST28111.1_2597	MOBP	Yes
CRMRs218	lcl|CP021764.1_prot_AST33053.1_2601	MOBP	Yes
PSI07	-	-	No
T51	-	-	No
T11	-	-	No
SL3175	lcl|CP022788.1_prot_AXW52807.1_1757	MOBP	Yes

	lcl|CP022788.1_prot_2117	MOBV	No
T98	lcl|CP022759.1_prot_AXV81667.1_1758	MOBP	Yes
	lcl|CP022759.1_prot_2118	MOBV	No
T12	-	-	No
SL3022	lcl|CP023016.1_prot_AXW60977.1_468	MOBP	No
SL2312	-	-	No
SL2064	-	-	No
T101	-	-	No
T95	-	-	No
T82	-	-	No
A2-HR	-	-	No
12D	lcl|CP001644.1_prot_ACS61995.1_682	MOBH	Yes
	lcl|CP001644.1_prot_ACS62082.1_769	MOBP	Plasmid
	lcl|CP001646.1_prot_ACS65975.1_4662	MOBH	Plasmid
	lcl|CP001647.1_prot_ACS66412.1_5099	MOBH	Plasmid
12J	lcl|CP001068.1_prot_ACD25607.1_440	MOBP	Yes
	lcl|CP001068.1_prot_ACD26658.1_1491	MOBH	Plasmid
	lcl|CP001068.1_prot_ACD27752.1_2585	MOBP	Plasmid
FDAARGOS_410	-	-	No
SN82F48	lcl|CP010799.2_prot_AJW44683.1_1559	MOBH	Yes
SN83A39	-	-	No

Table S4. Ralstonia spp. genomes coding T4CP and T4SS

Strain	T4SS		T4CP	ICE
Po82	No		Yes	No
UY031	No		Yes	No
UW163	3561100..3561765 [+], 666	VirB1	Yes	Plasmid
	3561819..3562112 [+], 294	VirB2		
	3562121..3562495 [+], 375	VirB3		
	3562504..3564993 [+], 2490	VirB4		
	3565048..3565779 [+], 732	VirB5		
	3565776..3565904 [+], 129	-		
	3565901..3566368 [+], 468	-		
	3566388..3567308 [+], 921	VirB6		
	3567568..3568344 [+], 777	VirB8		
	3568344..3569147 [+], 804	VirB9		
	3569144..3569851 [+], 708	-		
	3569844..3571088 [+], 1245	VirB10		
	3571085..3572131 [+], 1047	VirB11		
	3572128..3572484 [+], 357	VirD4		
	3572481..3574292 [+], 1812	VirD4		
IBSBF1503	No		Yes	No
RS 488	No		Yes	No
RS 489	No		Yes	No
CFBP2957	No		Yes	No
UW555	No		Yes	No
K60	No		Yes	No
GMI1000	2781912..2783189 [-], 1278	TrbI	Yes	Yes
	2783192..2784196 [-], 1005	TrbG		
	2784193..2784897 [-], 705	TrbF		
	2784928..2786301 [-], 1374	TrbL		
	2786298..2786624 [-], 327	TrbJ		
	2786637..2787374 [-], 738	TrbJ		
	2787371..2789821 [-], 2451	TrbE		
	2789834..2790106 [-], 273	TrbD		
	2790103..2790486 [-], 384	TrbC		
	2790492..2791559 [-], 1068	TrbB		
	2791556..2792032 [-], 477	-		
	2792029..2794056 [-], 2028	VirD4		
OE1	No		Yes	No
FJAT-1458	No		Yes	No
EP1	No		Yes	No
FJAT-91	1320491..1321594 [+], 1104	VirD4	Yes	Yes
	1321933..1323465 [+], 1533	-		
	1323419..1324414 [+], 996	VirD4		
	1324411..1324875 [+], 465	-		
	1324872..1325912 [+], 1041	TrbB		
	1325909..1326286 [+], 378	TrbC		
	1326283..1326552 [+], 270	TrbD		
	1326563..1328980 [+], 2418	TrbE		
	1328977..1329741 [+], 765	TrbJ		
	1329753..1331135 [+], 1383	TrbL		
	1331148..1331852 [+], 705	TrbF		
	1331849..1332841 [+], 993	TrbG		

	1333525..1334133 [+], 609	TrbI		
CQPS-1	No		Yes	No
RSCM	No		Yes	No
FQY_4	1157675..1159678 [+], 2004	VirD4	Yes	Yes
	1159675..1160139 [+], 465	-		
	1160136..1161188 [+], 1053	TrbB		
	1161185..1161562 [+], 378	TrbC		
	1161559..1161828 [+], 270	TrbD		
	1161839..1164289 [+], 2451	TrbE		
	1164286..1165026 [+], 741	TrbJ		
	1165038..1166417 [+], 1380	TrbL		
	1166442..1167146 [+], 705	TrbF		
	1167143..1168147 [+], 1005	TrbG		
	1168150..1169439 [+], 1290	TrbI		
SEPPX05	No		Yes	No
SL3103	No		Yes	No
SL2330	No		Yes	No
T117	No		Yes	No
Rs-10-244	No		Yes	No
Rs-09-161	No		No	No
T78	3990018..3990371 [-], 354	VirD4	Yes	Plasmid
	3990368..3991414 [-], 1047	VirB11		
	3991411..3992682 [-], 1272	VirB10		
	3992675..3993382 [-], 708	-		
	3993379..3994182 [-], 804	VirB9		
	3994182..3994958 [-], 777	VirB8		
	3995218..3996132 [-], 915	VirB6		
	3996152..3996619 [-], 468	-		
	3996616..3996744 [-], 129	-		
	3996741..3997475 [-], 735	VirB5		
	3997511..3998086 [-], 576	-		
	3998198..4000687 [-], 2490	VirB4		
	4000698..4001057 [-], 360	VirB3		
	4001081..4001374 [-], 294	VirB2		
	4001436..4002101 [-], 666	VirB1		
T25	No		Yes	No
SL3755	No		Yes	No
SL3730	No		Yes	No
SL2729	No		Yes	No
T110	No		Yes	No
T60	No		Yes	No
T42	No		Yes	No
SL3882	No		Yes	No
SL3822	No		Yes	No
SL3300	No		Yes	No
HA4-1	1744917..1746911 [+], 1995	VirD4	Yes	Yes
	1746908..1747372 [+], 465	-		
	1747369..1748409 [+], 1041	TrbB		
	1748406..1748783 [+], 378	TrbC		
	1748780..1749049 [+], 270	TrbD		
	1749060..1751510 [+], 2451	TrbE		
	1751507..1752235 [+], 729	TrbJ		
	1752247..1753623 [+], 1377	TrbL		

	1753636..1754340 [+], 705	TrbF		
	1754337..1755341 [+], 1005	TrbG		
	1755344..1756636 [+], 1293	TrbI		
	5873829..5874494 [+], 666	VirB1		Plasmid
	5874556..5874849 [+], 294	VirB2		
	5874873..5875232 [+], 360	VirB3		
	5875243..5877732 [+], 2490	VirB4		
	5878455..5879189 [+], 735	VirB5		
	5879186..5879314 [+], 129	-		
	5879311..5879778 [+], 468	-		
	5879798..5880712 [+], 915	VirB6		
	5880709..5880825 [+], 117	-		
	5880972..5881748 [+], 777	VirB8		
	5881748..5882551 [+], 804	VirB9		
	5882548..5883255 [+], 708	-		
	5883248..5884519 [+], 1272	VirB10		
	5884516..5885562 [+], 1047	VirB11		
	5885559..5885912 [+], 354	VirD4		
	5885912..5887726 [+], 1815	VirD4		
KACC10709	No		Yes	
KACC 10722	No		Yes	
CMR15	5576435..5577457 [-], 1023	VirB11	No	Plasmid
	5577469..5578695 [-], 1227	VirB10		
	5578697..5579491 [-], 795	VirB9		
	5579517..5580194 [-], 678	VirB8		
	5580191..5581048 [-], 858	VirB6		
	5581060..5581446 [-], 387	-		
	5581439..5581648 [-], 210	VirB7		
	5581694..5582356 [-], 663	VirB5		
	5582359..5585028 [-], 2670	VirB4		
	5585015..5585317 [-], 303	VirB3		
	5585314..5585688 [-], 375	VirB2		
RS 476	2781931..2783208 [-], 1278	TrbI	Yes	Yes
	2783211..2784215 [-], 1005	TrbG		
	2784212..2784916 [-], 705	TrbF		
	2784947..2786320 [-], 1374	TrbL		
	2786317..2786643 [-], 327	TrbJ		
	2786656..2787393 [-], 738	TrbJ		
	2787390..2789840 [-], 2451	TrbE		
	2789853..2790125 [-], 273	TrbD		
	2790122..2790505 [-], 384	TrbC		
	2790511..2791578 [-], 1068	TrbB		
	2791575..2792051 [-], 477	-		
	2792048..2794075 [-], 2028	VirD4		
CRMRs218	2783460..2784464 [-], 1005	TrbG	Yes	Yes
	2784461..2785165 [-], 705	TrbF		
	2785196..2786569 [-], 1374	TrbL		
	2786566..2786892 [-], 327	TrbJ		
	2786905..2787642 [-], 738	TrbJ		
	2787639..2790089 [-], 2451	TrbE		
	2790102..2790374 [-], 273	TrbD		
	2790371..2790754 [-], 384	TrbC		
	2790760..2791827 [-], 1068	TrbB		

	2791824..2792300 [-], 477	-		
	2792297..2794324 [-], 2028	VirD4		
YC40M	2534716..2536005 [-], 1290	TrbI	Yes	Yes
	2536008..2537012 [-], 1005	TrbG		
	2537009..2537713 [-], 705	TrbF		
	2537738..2539117 [-], 1380	TrbL		
	2539129..2539869 [-], 741	TrbJ		
	2539866..2542316 [-], 2451	TrbE		
	2542327..2542596 [-], 270	TrbD		
	2542593..2542970 [-], 378	TrbC		
	2542967..2544019 [-], 1053	TrbB		
	2544016..2544480 [-], 465	-		
	2544477..2546480 [-], 2004	VirD4		
PSI07	No		Yes	No
T51	No		Yes	No
T11	No		Yes	No
SL3175	No		Yes	No
T98	No		Yes	No
T12	No		Yes	No
SL3022	No		Yes	No
SL2312	No		Yes	No
SL2064	No		Yes	No
T101	No		Yes	No
T95	No		Yes	No
T82	No		Yes	No
A2-HR	No		Yes	No
12D	5056677..5058566 [+], 1890	TraG	Yes	Plasmid
	5058609..5059235 [+], 627	-		
	5059232..5059870 [+], 639	-		
	5060030..5061031 [+], 1002	TrbB		
	5060973..5061332 [+], 360	TrbC		
	5061348..5061668 [+], 321	TrbD		
	5061662..5064196 [+], 2535	TrbE		
	5064189..5064950 [+], 762	TrbJ		
	5064981..5065754 [+], 774	TrbJ		
	5065872..5067194 [+], 1323	-		
	5067247..5067537 [+], 291	-		
	5067611..5067769 [+], 159	-		
	5067766..5069706 [+], 1941	-		
	5069906..5070604 [+], 699	TrbF		
	5070604..5071371 [+], 768	TrbG		
	5071368..5072702 [+], 1335	TrbI		
	5072708..5074228 [+], 1521	VirB9		
12J	1609469..1611325 [-], 1857	TraG	Yes	ICE
	1611322..1612584 [-], 1263	-		
	1612589..1613659 [-], 1071	TrbL		
	1613671..1614462 [-], 792	TrbJ		
	1614519..1616966 [-], 2448	TrbE		
	1616960..1617289 [-], 330	TrbD		
	1617292..1617636 [-], 345	TrbC		
	1617649..1618698 [-], 1050	TrbB		
	1619120..1619833 [+], 714	TrbF		
	1619830..1620711 [+], 882	TrbG		

	1620722..1621933 [+], 1212	TrbI		
	1621926..1622726 [+], 801	TrbN		
	1622723..1623973 [+], 1251	-		
	1623970..1624446 [+], 477	TrbN		
	1624464..1624988 [+], 525	-		
	1624985..1626676 [+], 1692	-		
	1627094..1627399 [+], 306	-		
	1627401..1628654 [+], 1254	-		
	1628656..1630242 [+], 1587	TraJ_I		
	2757009..2759027 [+], 2019	VirD4	Yes	Plasmid
	2759024..2759488 [+], 465	-		
	2759485..2760522 [+], 1038	TrbB		
	2760519..2760911 [+], 393	TrbC		
	2760908..2761192 [+], 285	TrbD		
	2761212..2763689 [+], 2478	TrbE		
	2763686..2764441 [+], 756	TrbJ		
	2764454..2765806 [+], 1353	TrbL		
	2765825..2766529 [+], 705	TrbF		
	2766526..2767521 [+], 996	TrbG		
	2767524..2768795 [+], 1272	TrbI		
FDAARGOS_410	No		Yes	No
SN82F48	1648358..1648906 [+], 549	Tfc2	Yes	Yes
	1648903..1649550 [+], 648	Tfc2		
	1649560..1650279 [+], 720	Tfc3		
	1650264..1650884 [+], 621	Tfc4		
	1650881..1651414 [+], 534	Tfc5		
	1651423..1653579 [+], 2157	Tfc6		
	1653576..1654325 [+], 750	Tfc8		
	1691724..1692089 [+], 366	Tfc9		
	1692086..1692325 [+], 240	-		
	1692348..1692722 [+], 375	Tfc10		
	1692735..1693145 [+], 411	Tfc11		
	1693142..1693834 [+], 693	Tfc12		
	1693831..1694745 [+], 915	Tfc13		
	1694735..1696168 [+], 1434	Tfc14		
	1696149..1696583 [+], 435	Tfc15		
	1696583..1699471 [+], 2889	Tfc16		
	1699485..1700228 [+], 744	-		
	1700424..1700918 [+], 495	-		
	1701083..1701532 [+], 450	Tfc24		
	1701529..1702473 [+], 945	Tfc23		
	1702484..1703890 [+], 1407	Tfc22		
	1703887..1704240 [+], 354	-		
	1704256..1705779 [+], 1524	Tfc19		
SN83A39	No		Yes	No

Table S5. *Ralstonia* spp. genomes used in this work.

Strain	Assembly	Status	Phylotype	Origin
Ralstonia solanacearum (phylotype IIA and IIB)				
Po82	GCA_000215325.1	Complete Genome	IIB	Mexico
UY031	GCA_001299555.1	Complete Genome	IIB	Uruguay
UW163	GCA_001587135.1	Complete Genome	IIB	Peru
IBSBF1503	GCA_001587155.1	Complete Genome	IIB	Brazil
RS488	GCA_002501565.1	Complete Genome	II	Brazil
RS489	GCA_002549815.1	Complete Genome	II	Brazil
CFBP2957	GCA_000197855.1	Complete Genome	IIA	French West Indies
MolK2	GCA_000212635.2	Draft	IIB	Filipina
P673	GCA_000525615.1	Draft	IIB	USA
23-10BR	GCA_000749995.1	Draft	IIB	Brazil
NCPPB 282	GCA_000750575.1	Draft	IIB	Colombia
POPS2	GCA_000750585.1	Draft	IIB	China
CIP120	GCA_001644795.1	Draft	IIA	Peru
P597	GCA_001644805.1	Draft	IIA	USA
CFBP6783	GCA_001644815.1	Draft	IIB	Martinica
UW491	GCA_001696845.1	Draft	II	Colombia
UW24	GCA_001696855.1	Draft	II	Israel
UW365	GCA_001696865.1	Draft	II	China
UW551	GCA_001696875.1	Complete Genome	IIB	USA
UW25	GCA_002251695.1	Draft	II	USA
K60	GCA_000285815.1	Complete Genome	II	USA
Y45	GCA_000223115.2	Draft	IIB	China
IPO1609	GCA_001050995.1	Draft	IIB	Netherlands
CFIA906	GCA_000710135.3	Draft	II	-
NCPPB 909	GCA_000710695.1	Draft	IIB	Colombia
B50	GCA_000825785.2	Draft	IIA	Peru
CIP417	GCA_000825825.2	Draft	IIB	Filipinas
Grenada 9-1	GCA_000825845.2	Draft	IIA	Granada
CFBP1416	GCA_000825925.2	Draft	IIB	Costa Rica

CFBP7014	GCA_001373255.1	Draft	IIB	Trindade
IBSBF1900	GCA_001373275.1	Draft	IIA	Brazil
RS2	GCA_001373295.1	Draft	IIB	India
GEO_99	GCA_002029865.1	Draft	-	USA
GEO_57	GCA_002029885.1	Draft	-	USA
GEO_96	GCA_002029895.1	Draft	-	USA
GEO_6	GCA_002894765.1	Draft	-	USA
GEO_304	GCA_002894775.1	Draft	-	USA
GEO_81	GCA_002894785.1	Draft	-	USA
GEO_230	GCA_002894795.1	Draft	-	USA
GEO_55	GCA_002894845.1	Draft	-	USA
UW181	GCA_001373315.1	Draft	IIA	Venezuela
58_RSOL	GCA_001065525.1	Draft	-	USA
UW179	GCA_000825805.2	Draft	IIA	Colombia
CFBP3858	GCA_001373335.1	Draft	IIB	Netherlands
Ralstonia pseudosolanacearum (Ralstonia solanacearum phylotype I/III*)*				
GMI1000	GCA_000009125.1	Complete Genome	I	French Guiana
OE1-1	GCA_001879565.1	Complete Genome	I	Japan
FJAT-1458	GCA_001887535.1	Complete Genome	I	China
EP1	GCA_001891105.1	Complete Genome	I	China
FJAT-91	GCA_002155245.1	Complete Genome	I	China
CQPS-1	GCA_002220465.1	Complete Genome	I	China
RSCM	GCA_002894285.1	Complete Genome	I	China
FQY_4	GCA_000348545.1	Complete Genome	I	China
Rs-10-244	GCA_000671315.1	Complete Genome	I	India
SEPPX05	GCA_002162015.1	Complete Genome	I	China
Rs-09-161	GCA_000671335.1	Complete Genome	I	India
SL3103	GCA_003515205.1	Complete Genome	I	Korea
SL2330	GCA_003515225.1	Complete Genome	I	Korea
T117	GCA_003515245.1	Complete Genome	I	Korea
T78	GCA_003515285.1	Complete Genome	I	Korea
T25	GCA_003515305.1	Complete Genome	I	Korea

SL3755	GCA_003515345.1	Complete Genome	I	Korea
SL3730	GCA_003515365.1	Complete Genome	I	Korea
SL2729	GCA_003515405.1	Complete Genome	I	Korea
T110	GCA_003515465.1	Complete Genome	I	Korea
T60	GCA_003515545.1	Complete Genome	I	Korea
T42	GCA_003515565.1	Complete Genome	I	Korea
SL3882	GCA_003515585.1	Complete Genome	I	Korea
SL3822	GCA_003515605.1	Complete Genome	I	Korea
SL3300	GCA_003515625.1	Complete Genome	I	Korea
HA4-1	GCF_003999725.1	Complete Genome	I	China
P781	GCA_001644865.1	Draft	I	USA
KACC10709	GCA_001708525.1	Complete Genome	I	Korea
KACC 10722	GCA_001586135.1	Complete Genome	IIB	Korea
CaRs-Mep	GCA_001855495.1	Draft	I	India
PSS190	GCA_001870825.1	Draft	I	Taiwan
PSS216	GCA_001876975.1	Draft	I	Taiwan
RD15	GCA_001854265.1	Draft	-	Taiwan
PSS1308	GCA_001870805.1	Draft	-	Taiwan
SD54	GCA_000430925.2	Draft	I	China
PSS4	GCA_001876985.1	Draft	I	Taiwan
BBAC-C1	GCA_001920895.1	Draft	I	China
FJAT-452	GCA_001920905.1	Draft	I	China
CMR15	GCA_000427195.1	Complete Genome	III	Cameroon
CFBP3059	GCA_001644855.1	Draft	III	Burkina Faso
Bg07	GCA_003256445.1	Draft	I	-
Rs-T02	GCA_001484095.1	Draft	I	China
Cq01	GCA_003256425.1	Draft	I	-
Fm03	GCA_003256405.1	Draft	I	-
RS 476	GCA_003595305.1	Complete Genome	I	Brazil
CRMRs218	GCF_003612975.1	Complete Genome	I	Brazil
YC40M	GCA_001663415.1	Complete Genome	I	China

Ralstonia syzygii (*Ralstonia solanacearum* phlylotype IV)				
PSI07 (subsp. *indonesiensis*)	GCA_000283475.1	Complete Genome	IV	Indonesia
T51	GCA_003515145.1	Complete Genome	IV	Korea
T11	GCA_003515165.1	Complete Genome	IV	Korea
SL3175	GCA_003515185.1	Complete Genome	IV	Korea
T98	GCA_003515265.1	Complete Genome	IV	Korea
T12	GCA_003515325.1	Complete Genome	IV	Korea
SL3022	GCA_003515385.1	Complete Genome	IV	Korea
SL2312	GCA_003515425.1	Complete Genome	IV	Korea
SL2064	GCA_003515445.1	Complete Genome	IV	Korea
T101	GCA_003515485.1	Complete Genome	IV	Korea
T95	GCA_003515505.1	Complete Genome	IV	Korea
T82	GCA_003515525.1	Complete Genome	IV	Korea
A2-HR (subsp. *celebesensis*)	GCA_002012345.1	Complete Genome	IV	-
R229 (subsp. *celebesensis*)	PRJNA369602	Draft	IV	Malaysia
R24 (subsp. *syzygii*)	PRJNA53879	Draft	IV	-
Ralstonia pickettii				
12D	GCA_000023425.1	Complete Genome	-	USA
12J	GCA_000020205.1	Complete Genome	-	USA
FDAARGOS_410	GCA_002393485.1	Complete Genome	-	-
5_7_47FAA	GCA_000165085.1	Draft	-	Canada
52	GCA_002849525.1	Draft	-	-
ATCC 27511	GCA_000743455.1	Draft	-	-
H2Cu5	GCA_001699815.1	Draft	-	Portugal
H2Cu2	GCA_001699795.1	Draft	-	Portugal
ICMP-8657	GCA_002516395.2	Draft	-	Germany
NBRC 102503	GCA_001544155.1	Draft	-	-
OR214	GCA_000372665.1	Draft	-	-
DTP0602	GCA_000471925.1	Draft	-	-
Ralstonia mannitolilytica				-
SN82F48	GCA_000954135.2	Complete Genome	-	-
SN83A39	GCA_001628775.1	Complete Genome	-	-

Table S6. Conserved proteins used to identify Integrative and Conjugative Elements

Protein name	Function	Genbank Protein ID
integrase-tyrosine recombinase protein	Integration/Excision	CAD16329.1
traG	Conjugative transfer protein	CAD16293.1
trbB	Conjugative transfer protein	CAD16291.1
trbC	Conjugative transfer protein	CAD16290.1
trbD	Conjugative transfer protein	CAD16289.1
trbE	Conjugative transfer protein	CAD16288.1
trbJ	Conjugative transfer protein	CAD16287.1
trbL	Conjugative transfer protein	CAD16285.1
trbF	Conjugative transfer protein	CAD16284.1
trbG	Conjugative transfer protein	CAD16283.1
trbI	Conjugative transfer protein	CAD16282.1
traR	Conjugative transfer protein	CAD16295.1
P-type conjugative transfer protein TrbG	Conjugative transfer protein	ACD27778.1
Conjugal transfer protein	Conjugative transfer protein	ACD27777.1
P-type conjugative transfer protein TrbL	Conjugative transfer protein	ACD27776.1
P-type conjugative transfer protein TrbJ	Conjugative transfer protein	ACD27775.1
AAA ATPase	Integration/Excision	ACD27774.1
putative conjugal transfer TrbD transmembrane protein	Conjugative transfer protein	ACD27773.1
Conjugal transfer protein TrbC	Conjugative transfer protein	ACD27772.1
P-type conjugative transfer ATPase TrbB	Conjugative transfer protein	ACD27771.1
TRAG family protein	Conjugative transfer protein	ACD27769.1
putative conjugal transfer TRAF transmembrane protein	Conjugative transfer protein	ACD27751.1
ParB domain protein nuclease	Plasmid-partitioning protein	ACD27739.1
integrase family protein	Integration/Excision	ACD27728.1
putative integrase	Integration/Excision	AGH83603.1
Plasmid replication initiator protein	Replication protein	AGH83624.1
parA	Plasmid-partitioning protein	AGH83625.1
traF	Conjugative transfer protein	AGH83628.1
Type IV secretory pathway, VirD2 components (relaxase)	Conjugative transfer protein	AGH83629.1
virD4	Conjugative transfer protein	AGH83643.1
trbB	Conjugative transfer protein	AGH83645.1
trbC	Conjugative transfer protein	AGH83646.1
trbD	Conjugative transfer protein	AGH83647.1
trbE	Conjugative transfer protein	AGH83648.1
trbJ	Conjugative transfer protein	AGH83649.1
trbL	Conjugative transfer protein	AGH83650.1
trbF	Conjugative transfer protein	AGH83651.1
trbG	Conjugative transfer protein	AGH83652.1
trbI	Conjugative transfer protein	AGH83653.1
type VI secretion protein	Conjugative transfer protein	AJW44689.1
integrase	Integration/Excision	AJW44686.1
relaxase	Conjugative transfer protein	AJW44683.1
conjugal transfer protein	Conjugative transfer protein	AJW44677.1
conjugal transfer protein	Conjugative transfer protein	AJW44676.1
conjugal transfer protein	Conjugative transfer protein	AJW44672.1
conjugal transfer protein	Conjugative transfer protein	AJW44671.1
conjugal transfer protein	Conjugative transfer protein	AJW44670.1

conjugal transfer protein	Conjugative transfer protein	AJW44667.1
conjugal transfer protein	Conjugative transfer protein	AJW44666.1
bacterial conjugation TrbI-like family protein	Conjugative transfer protein	ANH72262.1
P-type conjugative transfer protein TrbG	Conjugative transfer protein	ANH73545.1
virB8 family protein	Conjugative transfer protein	ANH72290.1
P-type conjugative transfer protein TrbL	Conjugative transfer protein	ANH74679.1
putative lipoprotein	Conjugative transfer protein	ANH72153.1
P-type conjugative transfer protein TrbJ	Conjugative transfer protein	ANH73042.1
AAA-like domain protein	Conjugative transfer protein	ANH73147.1
type IV secretory pathway, VirB3-like family protein	Conjugative transfer protein	ANH74466.1
trbC/VIRB2 family protein	Conjugative transfer protein	ANH72057.1
P-type conjugative transfer ATPase TrbB	Conjugative transfer protein	ANH75107.1
traM recognition site of TraD and TraG family protein	Conjugative transfer protein	ANH72914.1
integrase	Integration/Excision	ANH33607.1
Plasmid replication initiator protein	Replication protein	ANH33585.1
Chromosome (plasmid) partitioning protein ParA	Plasmid-partitioning protein	ANH33583.1
Type IV secretory pathway, VirD2 components (relaxase)	Conjugative transfer protein	ANH33580.1
Type IV secretion system protein VirD4	Conjugative transfer protein	ANH33568.1
Conjugative transfer protein TrbB	Conjugative transfer protein	ANH33566.1
Conjugative transfer protein TrbC	Conjugative transfer protein	ANH33565.1
Conjugative transfer protein TrbD	Conjugative transfer protein	ANH33564.1
Conjugative transfer protein TrbE	Conjugative transfer protein	ANH33563.1
Conjugative transfer protein TrbJ	Conjugative transfer protein	ANH33562.1
Conjugative transfer protein TrbL	Conjugative transfer protein	ANH33561.1
Conjugative transfer protein TrbF	Conjugative transfer protein	ANH33560.1
Conjugative transfer protein TrbG	Conjugative transfer protein	ANH33559.1
Conjugative transfer protein TrbI	Conjugative transfer protein	ANH33558.1
conjugal transfer protein TrbI	Conjugative transfer protein	ARS55748.1
P-type conjugative transfer protein TrbG	Conjugative transfer protein	ARS55747.1
conjugal transfer protein TrbF	Conjugative transfer protein	ARS55746.1
P-type conjugative transfer protein TrbL	Conjugative transfer protein	ARS55745.1
P-type conjugative transfer protein TrbJ	Conjugative transfer protein	ARS57913.1
conjugal transfer protein TrbE	Conjugative transfer protein	ARS55744.1
conjugal transfer protein TrbD	Conjugative transfer protein	ARS55743.1
conjugal transfer protein TrbC	Conjugative transfer protein	ARS55742.1
P-type conjugative transfer ATPase TrbB	Conjugative transfer protein	ARS55741.1
type VI secretion protein	Conjugative transfer protein	ARS55733.1
chromosome partitioning protein ParB	Conjugative transfer protein	ARS57910.1
RepA replication protein	Replication protein	ARS55729.1
integrase	Integration/Excision	ARS55712.1
integrase	Integration/Excision	AST28132.1
chromosome partitioning protein ParB	Plasmid-partitioning protein	AST28127.1
chromosome partitioning protein ParB	Plasmid-partitioning protein	AST28115.1
type VI secretion protein	Conjugative transfer protein	AST28111.1
entry exclusion lipoprotein TrbK	Conjugative transfer protein	AST28098.1
conjugal transfer protein TraG	Conjugative transfer protein	AST28097.1
trbB	Conjugative transfer protein	AST28095.1
conjugal transfer protein TrbC	Conjugative transfer protein	AST28094.1
conjugal transfer protein TrbD	Conjugative transfer protein	AST28093.1
conjugal transfer protein TrbE	Conjugative transfer protein	AST28092.1

trbJ	Conjugative transfer protein	AST28091.1
trbL	Conjugative transfer protein	AST28089.1
conjugal transfer protein TrbF	Conjugative transfer protein	AST28088.1
trbG	Conjugative transfer protein	AST28087.1
conjugal transfer protein TrbI	Conjugative transfer protein	AST28086.1
integrase	Integration/Excision	AST33072.1
RepA replication protein	Replication protein	AST33969.1
chromosome partitioning protein ParB	Plasmid-partitioning protein	AST33968.1
type VI secretion protein	Conjugative transfer protein	AST33053.1
trbB	Conjugative transfer protein	AST33037.1
conjugal transfer protein TrbC	Conjugative transfer protein	AST33036.1
conjugal transfer protein TrbD	Conjugative transfer protein	AST33035.1
conjugal transfer protein TrbE	Conjugative transfer protein	AST33034.1
trbJ	Conjugative transfer protein	AST33033.1
hypothetical protein	Conjugative transfer protein	AST33032.1
trbL	Conjugative transfer protein	AST33031.1
conjugal transfer protein TrbF	Conjugative transfer protein	AST33030.1
trbG	Conjugative transfer protein	AST33029.1
conjugal transfer protein TrbI	Conjugative transfer protein	AST33028.1
ISL3 family transposase ISRso15	Conjugative transfer protein	AST33026.1
conjugal transfer protein TrbI	Conjugative transfer protein	AZU56157.1
trbG	Conjugative transfer protein	AZU56156.1
conjugal transfer protein TrbF	Conjugative transfer protein	AZU56155.1
trbL	Conjugative transfer protein	AZU56154.1
trbJ	Conjugative transfer protein	AZU56153.1
conjugal transfer protein TrbE	Conjugative transfer protein	AZU56152.1
conjugal transfer protein TrbD	Conjugative transfer protein	AZU56151.1
conjugal transfer protein TrbC	Conjugative transfer protein	AZU56150.1
trbB	Conjugative transfer protein	AZU56149.1
conjugal transfer protein TraG	Conjugative transfer protein	AZU56147.1
entry exclusion lipoprotein TrbK	Conjugative transfer protein	AZU56146.1
type VI secretion protein	Conjugative transfer protein	AZU56142.1
chromosome partitioning protein ParB	Plasmid-partitioning protein	AZU58031.1
RepA replication protein	Replication protein	AZU58029.1
chromosome partitioning protein ParB	Plasmid-partitioning protein	AZU56134.1
integrase	Integration/Excision	AXV81690.1
site-specific DNA-methyltransferase	Maintenance	AXV81685.1
chromosome partitioning protein ParB	Plasmid-partitioning protein	AXV81679.1
RepA replication protein	Replication protein	AXV81673.1
chromosome partitioning protein ParB	Plasmid-partitioning protein	AXV81670.1
type VI secretion protein	Conjugative transfer protein	AXV81667.1
conjugal transfer protein TraG	Conjugative transfer protein	AXV81650.1
trbB	Conjugative transfer protein	AXV81648.1
conjugal transfer protein TrbC	Conjugative transfer protein	AXV81647.1
conjugal transfer protein TrbD	Conjugative transfer protein	AXV81646.1
conjugal transfer protein TrbE	Conjugative transfer protein	AXV81645.1
trbJ	Conjugative transfer protein	AXV81644.1
hypothetical protein	Conjugative transfer protein	AXV81643.1
trbL	Conjugative transfer protein	AXV81642.1
conjugal transfer protein TrbF	Conjugative transfer protein	AXV81641.1

trbG	Conjugative transfer protein	AXV81640.1
conjugal transfer protein TrbI	Conjugative transfer protein	AXV83135.1
integrase	Integration/Excision	AXW52827.1
site-specific DNA-methyltransferase	Maintenance	AXW52823.1
chromosome partitioning protein ParB	Plasmid-partitioning protein	AXW52817.1
RepA replication protein	Replication protein	AXW52812.1
ATPase	Conjugative transfer protein	AXW52810.1
chromosome partitioning protein ParB	Conjugative transfer protein	AXW54255.1
type VI secretion protein	Conjugative transfer protein	AXW52807.1
conjugal transfer protein TraG	Conjugative transfer protein	AXW52791.1
trbB	Conjugative transfer protein	AXW52789.1
conjugal transfer protein TrbC	Conjugative transfer protein	AXW52788.1
conjugal transfer protein TrbD	Conjugative transfer protein	AXW52787.1
conjugal transfer protein TrbE	Conjugative transfer protein	AXW52786.1
trbJ	Conjugative transfer protein	AXW52785.1
trbL	Conjugative transfer protein	AXW52783.1
conjugal transfer protein TrbF	Conjugative transfer protein	AXW52782.1
trbG	Conjugative transfer protein	AXW52781.1
conjugal transfer protein TrbI	Conjugative transfer protein	AXW54251.1
